# Outcome of tuberculosis treatment in HIV-positive adults diagnosed through active versus passive case-finding

**DOI:** 10.3402/gha.v8.27048

**Published:** 2015-03-27

**Authors:** Taye T. Balcha, Sten Skogmar, Erik Sturegård, Per Björkman, Niclas Winqvist

**Affiliations:** 1 Infectious Disease Research Unit, Department of Clinical Sciences Malmö, Lund University, Malmö, Sweden; 2 Ministry of Health, Addis Ababa, Ethiopia; 3 Clinical Microbiology, Regional and University Laboratories, Region Skåne, Sweden; 4 Regional Department of Infectious Disease Control and Prevention, Malmö, Sweden

**Keywords:** active case-finding, passive case-finding, TB, HIV, adverse treatment outcomes

## Abstract

**Background:**

The World Health Organization strongly recommends regular screening for tuberculosis (TB) in HIV-positive individuals.

**Objective:**

To compare the outcome of anti-tuberculosis treatment (ATT) in HIV-positive adults diagnosed with TB through active case-finding (ACF) or passive case-finding (PCF).

**Design:**

Antiretroviral therapy (ART)-naïve adults diagnosed with TB were included from two prospective cohort studies conducted in Ethiopia between September 2010 and March 2013. The PCF cohort was based at out-patient TB clinics, whereas participants in the ACF cohort were actively screened for TB by bacteriological sputum testing (smear microscopy, Xpert MTB/RIF assay, and liquid culture) without pre-selection on the basis of symptoms and signs. Outcomes of ATT were compared between participants in the two cohorts; characteristics at diagnosis and predictors of adverse outcomes were analysed.

**Results:**

Among 439 TB/HIV co-infected participants, 307 and 132 belonged to PCF and ACF cohorts, respectively. Compared with the ACF participants, hemoptysis, conjunctival pallor, bedridden status, and low mid upper-arm circumference (MUAC) were significantly more common in participants identified through PCF. Sputum smear-positivity rates among pulmonary TB cases were 44.2% and 21.1% in the PCF and ACF cohorts, respectively (*p*<0.001). Treatment success was ascertained in 247 (80.5%) of the participants in the PCF cohort and 102 (77.2%) of the participants in the ACF cohorts (*p*=0.223). Low MUAC (*p*=0.001) independently predicted mortality in the participants in both cohorts.

**Conclusion:**

Although patients identified through ACF had less advanced TB disease, ATT outcome was similar to the patients identified through PCF. To achieve a better outcome, case management in ACF strategy should be strengthened through enhanced patient-centred counselling and adherence support.

Co-infection with tuberculosis (TB) and HIV is common in low-income countries. Collaborative TB/HIV activities are needed to reduce the burden of TB in people living with HIV (PLHIV) (1). For individuals with TB/HIV co-infection, there are two routes of entry into care: via either TB or HIV clinics. Whereas TB clinics detect TB among patients who seek medical care with symptoms (passive case-finding; PCF), active case-finding (ACF) entails screening for active TB for certain population groups regardless of symptoms or clinical suspicion. With PCF strategy, a substantial proportion of active TB patients might die before seeking care or be missed even after they reach health facilities (2). Many TB patients also become contagious before diagnosis. Conversely, ACF could detect TB cases early and is consequently recommended by WHO for PLHIV (3, 4).

Although HIV testing has been efficiently implemented in TB clinics, TB case-finding among PLHIV is suboptimal. Most persons who receive their HIV diagnosis when presenting with TB have advanced disease at presentation, with a high risk of death (5). A challenge for TB/HIV integration in high-burden countries is the lack of effective diagnostic algorithms and tools for TB detection in PLHIV (5–7).

Sputum smear microscopy detects the most contagious cases; however, a majority of HIV-associated TB is missed by this method. The WHO TB symptom screening algorithm can be used to identify subsets of PLHIV in need of further TB investigations (8). Still, in routine care 20–28% of active TB patients were missed at initiation of ART (9, 10). Therefore, TB screening using more sensitive diagnostic tests prior to ART initiation has been recommended (11–13).

A systematic review of intensified TB screening among PLHIV in low-resource, high-burden countries has reported high rates of TB detection (14). Also, an ACF study conducted in Ethiopia at ART clinics at health centres found 17.9% previously undetected TB cases (15).

Early identification of TB in PLHIV would be expected to lead to improved survival, and reduced risk of TB transmission (16). Although facility-based ACF for TB among PLHIV detects early cases and may consequently improve survival (17–19), empirical evidence on the benefit of ACF for treatment outcome is scarce. A recent systematic review has shown similar treatment outcome between cases identified through PCF and ACF strategies (20). In this study, we hypothesised that early identification of TB in PLHIV through ACF would lead to lower rates of adverse treatment outcomes. We compared anti-TB treatment (ATT) outcome among HIV-positive adults receiving TB care at out-patient TB clinics (PCF cohort) to that of PLHIV diagnosed with TB through intensified case-finding (ACF cohort) at HIV clinics.

## Design

### Characteristics of study participants

Participants were identified from two cohort studies (15, 21) conducted in public health facilities providing integrated care for TB and HIV in Oromia region, Ethiopia.

#### PCF cohort

Between September 2010 and September 2012, we recruited participants consecutively at eight out-patient TB clinics (two hospitals and six health centres; *n*=1,116). These patients had been diagnosed with TB at in- or out-patient clinics at these health facilities, or at private clinics after self-presentation, and were referred for ATT to the study TB clinics. Diagnosis of TB was based on sputum smear microscopy, clinical criteria, and chest radiography in accordance with Ethiopian National Guidelines (22). The following inclusion criteria were applied: age 18 years or greater, residence in the clinic uptake area, written informed consent, and consent to HIV testing. We excluded persons who had received ATT for more than 2 weeks or had been treated for TB within the preceding 6 months, and all ART experienced patients.

At baseline, trained nurses evaluated participants using a structured questionnaire with details on socio-demographic characteristics, self-reported symptoms, physical findings, and basic laboratory tests including complete blood count (CBC) and CD4 cell count. Detailed description of the cohort was presented recently (21). For the current study, we included 307 TB/HIV co-infected participants.

#### ACF cohort

We recruited HIV-positive participants in a prospective cohort between October 2011 and March 2013 at five health centre HIV clinics. At baseline, participants underwent testing for TB irrespective of symptoms. Consecutive individuals (both in HIV care and new cases) were assessed for eligibility and included individuals (*n*=812) were screened for TB. The ACF cohort had the following inclusion criteria: provision of written informed consent, age 18 years or older, CD4 count ≤350 cells/mm^3^ and/or WHO clinical stage 4, and ART-naïve. Trained nurses collected information on social, demographic, and clinical characteristics, and blood for CBC and CD4 cell count was obtained. The participants submitted two spontaneously expectorated morning sputum samples for bacteriological testing (including liquid culture, Xpert MTB/RIF, and smear microscopy; reported previously) (15). From participants with peripheral lymphadenopathy, fine-needle aspirates were obtained for culture and Xpert MTB/RIF assay. A total of 132 TB/HIV co-infected participants were included in the ACF cohort.

### Definitions and determination of ATT outcome

A TB case was defined as a patient with a positive bacteriological test result for TB and/or who fulfilled Ethiopian national criteria for clinical TB (22), and who was prescribed ATT using WHO's Directly Observed Treatment Short course (DOTS) strategy.

Prospective evaluations were performed 2 and 6 or 8 months after enrolment for all participants. Treatment outcome was assessed at the end of therapy (6 or 8 months). Treatment success was defined as cure and/or treatment completion (23). Loss to follow-up, treatment failure, and death were categorised as adverse outcome. Participants transferred to other health facilities for care could not be evaluated for treatment outcome and were consequently categorised as having neither treatment success nor adverse treatment outcome.

### Statistical analysis

Anonymised data were entered into Excel files continuously and crosschecked with original hard copies. Data analysis was performed using IBM SPSS 20.0 (SPSS Inc., Chicago, IL, USA). Using Pearson's Chi-Square test, categorical data on social, demographic, clinical, and laboratory information were compared between the two groups. Comparisons of medians for scale variables without normal distribution were performed using non-parametric Mann-Whitney's U-test. Similar tests were performed to compare outcomes of ATT between participants in the two cohorts. The level of statistical significance was set at *p*<0.05.

### Ethical considerations

Both studies were approved by the National Research Ethics Review Committee at the Ministry of Science and Technology of Ethiopia and the Regional Ethical Review Board at Lund University, Sweden. Written informed consent was obtained from all participants; in the presence of a witness in the case of illiterate participants.

## Results

### Baseline characteristics of the study participants

Characteristics of the participants are shown in Table 1. Among the 307 PCF participants, the median age was 32 years, 151 (49.2%) were female, and the median CD4 cell count was 173 cells/mm^3^. Among the 132 ACF participants, the median age was 35 years, 64 (48.5%) were female, and the median CD4 cell count was 169 cells/mm^3^. Rural residence was more common in the ACF cohort (21.7% vs 10.5%; *p*=0.03).

**Table 1:** Comparison of baseline characteristics, signs, and symptoms stratified by tuberculosis case-finding

Categories	Variables	Description	ACF^a^ TB cases (*n*=132; %)	PCF^b^ TB cases (*n*=307; %)	*p*
Total cohort (%)	–	–	132 (30.1)	307 (69.9)	**–**
Baseline characteristics	Gender	Male	68 (51.5)	156 (50.8)	0.917
		Female	64 (48.5)	151 (49.2)	
	Age (years)	Median age	35 (28–44^c^)	32 (28–40^c^)	0.120
	Residence	Urban	101 (78.3)	274 (89.5)	0.03
		Rural	28 (21.7)	32 (10.5)	
	CD4 count (cells/mm^3^)	Median	169 (91–271^c^)^d^	173 (95–336^a^)	0.160
	Hemoglobin (g/dL)	Median	10.4 (9.1–11.9^c^)	10.7 (9.3–12.0^c^)	0.289
	Type of TB	Pulmonary	128 (97.0%)	224 (73.0)	<0.001
		Extrapulmonary	4 (3.0%)	83 (27.0)	
	Pulmonary TB smear status	Positive	27 (21.1)	99 (44.2)	<0.001
		Negative	101 (78.9)	125 (55.8)	
WHO symptoms	WHO symptom screen^e^	Positive	121 (92.4)	292 (95.7)	0.164
		Negative	10 (7.6)	13 (4.3)	
	Fever	Yes	86 (65.6)	307 (82.4)	<0.001
		No	37 (34.4)	54 (17.6)	
	Night sweats	Yes	95 (72.0)	256 (83.4)	<0.01
		No	40 (28.0)	51 (16.6)	
	Weight loss	Yes	109 (82.6)	254 (83.0)	0.891
		No	23 (17.4)	52 (17.0)	
	Cough	Yes	82 (62.1)	190 (62.1)	1.0
		No	50 (37.9)	116 (37.9)	
Other symptoms	Loss of appetite	Yes	85 (64.4)	249 (81.1)	<0.001
		No	47 (35.6)	58 (18.9)	
	Blood stained sputum	Yes	6 (4.5)	47 (15.4)	0.001
		No	126 (95.5)	258 (84.6)	
	Bed ridden	Yes	13 (9.9)	81 (26.4)	<0.001
		No	118 (90.1)	226 (73.6)	
Physical findings	Conjunctival pallor	Yes	34 (26.0)	118 (38.4)	0.012
		No	97 (74.0)	189 (61.6)	
	Peripheral lymphadenopathy	Yes	14 (10.6)	58 (19.0)	0.035
		No	118 (89.4)	248 (81.0)	
Biometric data	BMI^f^ (kg/m^2^)	Median	17.8 (16.2–19.7^c^)	17.5 (16.0–19.5^c^)	0.342
	MUAC^g^ (cms)	Median	21.0 (19.0–23.0^c^)	20.0 (19.0–22.0^c^)	0.033

a ACF: active case-finding; TB case detected through intensified screening regardless of symptoms.

b PCF: passive case-finding; TB case detected after a symptomatic patient came to a health facility.

c Interquartile range.

d CD4≤350 cells/mm^3^ inclusion criteria for ACF.

e WHO symptom screen positive: presence of current cough, fever, night sweats or weight loss.

f BMI: body mass index.

g MUAC: mid upper-arm circumference.

A total of 124 (94%) study participants in the ACF cohort were culture and/or Xpert MTB/RIF positive, whereas 8 (6%) were clinically diagnosed. In the PCF cohort, 208 (67.8%) were clinically diagnosed TB cases (*p*<0.001). In the PCF and ACF cohorts, 7 (2.3%) and 5 (3.8%), respectively, had a previous episode of TB.

The smear-positivity rate among pulmonary TB cases was higher in the PCF cohort than in the ACF cohort (44.2% vs 21.1%; *p*<0.001). The distribution of symptoms and physical findings showed some differences between participants in the two cohorts. Specifically, fever (82.4% vs 65.6%; *p*<0.001) and night sweats (83.4% vs 72.0%; *p*<0.01) were significantly more common among patients recruited through PCF than in the ACF cohort. Similarly, markers of disease severity like blood stained sputum (15.4% vs 4.5%; *p*=0.001), conjunctival pallor (38.4% vs 26.0%; *p*=0.012), bedridden state (26.4% vs 9.9%; *p*<0.001), and lower MUAC (*p*=0.033) were more common among participants in the PCF cohort than in the ACF cohort (Table 1).

### ART initiation

A total of 236 (68.4%) and 75 (52.4%) participants in the PCF and ACF cohorts started ART during the course of ATT, respectively. Additionally, 34 (23.4%) participants in the ACF cohort started ART before ATT initiation. No PCF participant started ART before ATT.

### Outcomes of ATT

In the PCF cohort, 247 (80.5%) participants had treatment success, whereas 37 (12%) had adverse outcomes [20 (6.5%) were lost to follow-up and 17 (5.5%) died]. Treatment outcome in the PCF cohort could not be evaluated in 23 (7.5%) participants as they were transferred to other health facilities. Likewise, 102 (77.2%) participants in the ACF cohort had treatment success and 22 (16.7%) had adverse treatment outcomes [12 (9.1%) were lost to follow-up and 10 (7.6%) died]. Eight (6.1%) participants in the ACF were transferred to other health facilities (Table 2). Of seven previously treated participants in the PCF cohort, four had treatment success, whereas two participants died and one was lost to follow-up. Among five previously treated ACF participants, four had treatment success and one participant was transferred out.

**Table 2:** Comparison of treatment outcomes between TB cases detected through active and passive case-finding

TB treatment outcome	ACF^a^ TB cases	PCF^b^ TB cases	*p* c
Total cohort	132	307	–
Cured or completed treatment, *n* (%)	102 (77.2)	247 (80.5)	0.223
Lost to follow-up, *n* (%)	12 (9.1)	20 (6.5)	
Died, *n* (%)	10 (7.6)	17 (5.5)	
Transfer of care, *n* (%)	8 (6.1)	23 (7.5)	–

a ACF: active case-finding; TB case detected through intensified screening regardless of symptoms.

b PCF: passive case-finding; TB case detected after a symptomatic patient came to a health facility.

c Cured or completed treatment versus loss to follow-up or death.

A sub-analysis of participants with adverse outcomes in the ACF cohort showed that 10/22 (45.5%) TB patients never started ATT (all culture-positive). When these participants were tentatively included in the analysis of treatment outcome, there was no significant difference between the participants in the two cohorts (*p*=0.223). An analysis restricted to participants in the ACF cohort that initiated ART also showed no significant difference in ATT outcome with regard to the sequence of TB and HIV treatment initiation.

### Factors associated with adverse treatment outcomes

Adverse treatment outcomes in TB cases detected through PCF were associated with lower body mass index (BMI) (median 16.9 vs 17.6 kg/m^2^; *p*=0.015) and lower MUAC (median 19 vs 21 cm; *p*=0.001). Median baseline CD4 cell count ≤100 cells/mm^3^ (*p*=0.023), lower BMI (median 16.3 vs 17.8 kg/m^2^; *p*=0.036) and lower MUAC (20 vs 22 cm; *p*=0.042) were associated with adverse treatment outcomes among subjects in the ACF cohort (Table 3).

**Table 3:** Characteristics of TB cases detected through active and passive case-finding stratified by treatment outcomes

		ACF cohort^a^			PCF cohort^b^		
		
Patient characteristics	Variables	TS^c^ (%)	AO^d^ (%)	*p*	TS (%)	AO (%)	*p*
Gender	Male	54 (52.9)	10 (51.9)	0.639	124 (50.0)	22 (53.7)	0.737
	Female	48 (47.1)	12 (48.1)		124 (50.0)	19 (46.3)	
Age (years)	Median age	36 (28–45^e^)	30 (28–36^e^)	0.225	33 (28–40^e^)	30 (27–40^e^)	0.659
Residence^f^	Urban	79 (79.0)	15 (71.4)	0.564	223 (89.9)	35 (87.5)	0.584
	Rural	21 (21.0)	6 (28.6)		25 (10.1)	5 (12.5)	
CD4 count (cells/mm^3^)	Median CD4 count	170 (98–278^e^)	102 (77–260^e^)	0.379	178 (105–344^e^)	173 (70–320^e^)	0.598
CD4 cell strata (cells/mm^3^)	>200	38 (37.3)	9 (40.9)	0.023	113 (46.4)	17 (41.5)	0.227
	101–200	37 (36.3)	2 (9.1)		73 (29.4)	9 (22.0)	
	≤100	27 (26.5)	11 (50.0)		60 (24.2)	15 (36.6)	
Hemoglobin (g/dL)	Median hemoglobin	10.5 (9.3–12.0^e^)	9.7 (8.4–11.0^e^)	0.147	10.7 (9.3–12.0^e^)	11.2 (9.7–12.2^e^)	0.444
Type of TB	Pulmonary	99 (97.1)	21 (95.5)	0.547	179 (72.2)	33 (80.5)	0.341
	Extrapulmonary	3 (2.9)	1 (4.5)		69 (27.8)	8 (19.5)	
Smear status	Positive	18 (18.2)	7 (33.3)	0.142	79 (44.1)	15 (45.5)	1.0
	Negative	81 (81.8)	14 (66.7)		100 (55.9)	118 (54.5)	
BMI (kg/m^2^)	Median BMI	17.8 (16.5–19.8^e^)	16.3 (14.8–19.0^e^)	0.036	17.6 (16.2–19.6^e^)	16.9 (15.3–18.1^e^)	0.015
MUAC (cms)	Median MUAC	21.5 (19.0–23.0^e^)	20.0 (18.5–22.0^e^)	0.042	21.0 (19.0–23.0^e^)	19.0 (18.0–21.0^e^)	0.001
WHO symptom screen	Positive	92 (91.1)	21 (95.5)	0.689	233 (94.7)	41 (100)	0.226
	Negative	9 (8.9)	1 (4.5)		13 (5.3)	0 (0)	

a ACF: active case-finding; TB case detected through intensified screening regardless of symptoms.

b PCF: passive case-finding; TB case detected after a symptomatic patient came to a health facility

c TS: treatment success which includes TB patients who were cured or completed treatment.

d AO: adverse TB treatment outcome which includes loss to follow-up or death.

e Interquartile range.

f Residence: missing in 11 study participants in the ACF cohort.

## Discussion

We compared outcomes of ATT among HIV-positive ART-naïve adults treated at TB clinics in Ethiopia with regard to entry into care – either through active TB case-finding in HIV clinics, or passively referred for ATT. Although participants diagnosed through ACF had characteristics of less advanced TB, the outcome of ATT was similar to that of passively detected cases. This underscores both the importance and the challenge of ACF particularly for achieving optimal treatment success.

Evaluations of ACF are inherently difficult to perform, since randomisation of participants into ACF or PCF strategy is impossible. A feasibility study in South Africa showed high treatment success rate among TB patients identified through ACF using a mobile HIV/TB clinic (18). Nevertheless, another comparative study conducted in a similar setting and a recent systematic review failed to show improvement in outcome of ACF strategy (20, 24).

The rationale for ACF for TB among PLHIV in endemic regions is obvious due to the high prevalence of active TB and the severe consequences of unrecognised TB (17, 18). In this study, we did not find a difference in ATT outcomes between participants in the two cohorts. Our data indicate the importance of linking patients to care after submission of samples for testing. Although we did not measure adherence, other studies have suggested worse ATT adherence among patients diagnosed through ACF (14). In fact, the ACF cohort comprised more patients with rural residence, a factor that has been associated with lower adherence in other studies (25).

In the ACF cohort, a set of bacteriological methods in HIV-positive individuals without pre-selection on the basis of symptoms identified TB in 132 of 812 (16.3%) participants, confirming a high yield of ACF among PLHIV (15). Ten (7.6%) of the TB patients in our ACF cohort never started ATT. Failure to start ATT was largely due to early loss to follow-up or death which occurred between submissions of samples and result delivery. The higher rate of early loss to follow-up in the ACF cohort was in concordance with the findings from community-based ACF in Cambodia (26). Consistent with these findings, it is possible that rates of loss to follow-up are higher in patients recruited through ACF (14, 19). On the contrary, TB patients identified through PCF strategy were immediately linked to treatment. It is also likely that a large proportion of TB/HIV co-infected persons in the community are not recognised as TB cases; hence, our PCF cohort only included a subset of the real population of such individuals.

Less advanced disease characteristics were recorded among TB cases detected actively compared with PCF cases. Biometric measurements indicating disease severity, like BMI and MUAC, were lower in the PCF study participants. This finding is consistent with a similar report from South Africa, showing that ACF identified subjects with minor or early symptoms and signs of TB (24). Further, 78.9% of pulmonary TB cases in the ACF cohort were smear-negative. It is likely that some of those cases would have progressed to smear-positivity; which suggests that ACF could help reducing the burden of contagious TB in the community (27). The advanced disease characteristics in the PCF study participants also implies that TB diagnosis in this subgroup of patients generally occurs later, compared with those who enter into care via HIV clinics (28).

The WHO 3Is policy (Intensified TB case-finding; Isoniazid preventive therapy; and Improved TB infection control) has been vital in identifying a substantial proportion of TB cases among PLHIV (29); yet its implementation has not been uniform (6, 7). A report from South Africa showed that 87% of TB cases occurring during the first year on ART could have been detected at baseline using sputum culture (16).

In the ACF cohort, 75 (52.4%) of the TB patients initiated ART during the course of TB treatment. Of particular note, 34 (23.4%) started ART prior to TB treatment in the ACF cohort, mainly due to onerous culture test result delivery. However, ART initiation before ATT did not significantly increase the risk of mortality in our population.

Adverse TB treatment outcome in actively detected cases was associated with CD4 count ≤100 cells/mm^3^ and low BMI and MUAC. Likewise, low BMI and MUAC were also associated with adverse treatment outcome in PCF cases. Our results support the utility of MUAC as a predictor for mortality in HIV-positive individuals with TB (30).

Participants for this study were recruited from two cohorts of HIV-positive individuals with overlapping periods of enrolment, using similar recording of baseline characteristics and follow-up. Further, patient management, including ART initiation, was at the discretion of the attending clinicians for both cohorts.

This study has some limitations. Whereas the ACF cohort had eligibility criteria of baseline CD4 count ≤350 cells/mm^3^ and/or WHO stage 4, all ART-naïve TB patients irrespective of CD4 count and WHO stage were recruited in the PCF cohort. Consequently, 73/307 (23.8%) of the PCF cohort had CD4 count >350 cells/mm^3^. Additionally, methods for TB diagnosis were different in the two cohorts. In the ACF cohort, all participants underwent bacteriological TB testing, whereas national guidelines were used for TB diagnosis in the PCF cohort. Hence, it is possible that some of the clinically diagnosed PCF cases may have had other conditions than TB (31). Whereas a proportion of participants in the PCF cohort were diagnosed by physicians, the ACF participants were largely identified by non-physician clinicians which might have contributed to the higher proportion of extrapulmonary cases in the PCF cohort. Finally, although the two cohorts were recruited from similar settings, potential differences in socio-demographic characteristics might have obscured any difference in ATT outcomes between the two groups.

## Conclusion

Although ACF in PLHIV led to detection of TB cases with less advanced clinical characteristics, ATT outcome was similar to that in PCF subjects. A proportion of patients identified through ACF never started ATT. As the principal goals of early diagnosis are early treatment initiation and successful outcome, targeted ACF using effective diagnostic tools should be coupled with intensive patient-centred counselling. We recommend further epidemiological studies that investigate effectiveness and costs associated with ACF at health facilities and communities with high burden of TB and HIV.
